# Role of Methionine Adenosyltransferase Genes in Hepatocarcinogenesis

**DOI:** 10.3390/cancers3021480

**Published:** 2011-03-24

**Authors:** Komal Ramani, José M. Mato, Shelly C. Lu

**Affiliations:** 1 Division of Gastroenterology and Liver Diseases, USC Research Center for Liver Diseases, Southern California Research Center for Alcoholic Liver and Pancreatic Diseases & Cirrhosis, Keck School of Medicine USC, Los Angeles, California 90033, USA; E-Mail: kramani@usc.edu; 2 CIC bioGUNE, Centro de Investigación Biomédica en Red de Enfermedades Hepáticas y Digestivas (Ciberehd), Technology, Park of Bizkaia, 48160 Derio, Bizkaia, Spain; E-Mail: director@cicbiogune.es

**Keywords:** methionine adenosyltransferase (MAT), hepatocellular carcinoma (HCC), S-adenosylmethionine (AdoMet)

## Abstract

Hepatocellular carcinoma (HCC) is the most common primary malignant tumor of the liver. Detection of HCC can be difficult, as most of the patients who develop this tumor have no symptoms other than those related to their longstanding liver disease. There is an urgent need to understand the molecular mechanisms that are responsible for the development of this disease so that appropriate therapies can be designed. Methionine adenosyltransferase (MAT) is an essential enzyme required for the biosynthesis of S-adenosylmethionine (AdoMet), an important methyl donor in the cell. Alterations in the expression of MAT genes and a decline in AdoMet biosynthesis are known to be associated with liver injury, cirrhosis and HCC. This review focuses on the role of MAT genes in HCC development and the scope for therapeutic strategies using these genes.

## 1. Introduction

Hepatocellular carcinoma (HCC) is the fifth most common cancer worldwide and its incidence in the United States and other countries has been steadily increasing over the past 25 years [1,2]. The primary risk factors for HCC include infection with hepatitis B and hepatitis C viruses, and long-term exposure to aflatoxin [3]. In the United States, chronic alcoholism leading to chronic liver disease is a significant risk factor [4]. The prognosis for HCC is poor and lack of good diagnostic markers and treatment options have rendered this disease a major health problem [5].

At the molecular level, several proteins have been identified as de-regulated during HCC. These include p53, Retinoblastoma (Rb), Insulin-like growth factor receptor 1 (IGFR-1), β-catenin, and cyclin D1 [6]. Other signaling pathways relevant in HCC development include Phosphatidyl inositol-3 Kinase (PI3-K) and c-jun N-terminal kinase (JNK) [7,8]. This paper reviews the role of methionine adenosyltransferase (MAT) genes in the development and possible treatment of HCC.

## 2. Hepatic Methionine Metabolism

**Figure 1.**
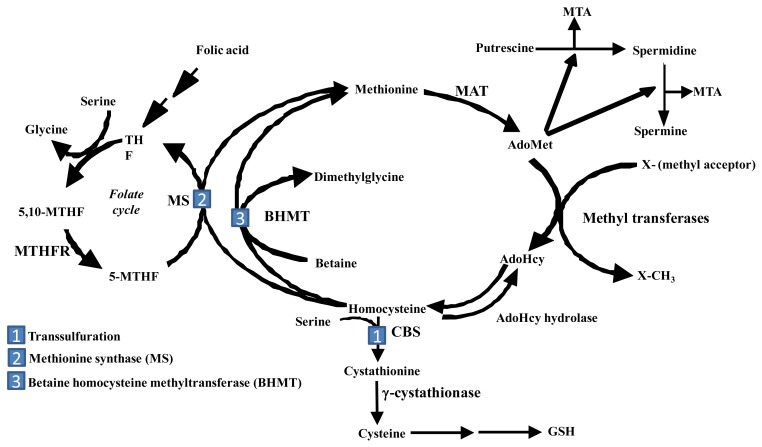
Methionine metabolism in liver. The first step in methionine metabolism is catalyzed by methionine adenosyltransferase (MAT), generating S-adenosylmethionine (AdoMet), which is converted to AdoHcy during transmethylation reactions. AdoHcy hydrolase catalyzes the reversible hydrolysis of AdoHcy to yield homocysteine and adenosine. In the liver, homocysteine can undergo three metabolic pathways (labeled as 1, 2 and 3). First is the transsulfuration pathway, which converts homocysteine to cysteine through a two-step process consisting of two vitamin B_6_ dependent enzymes, cystathionine β-synthase (CBS) and γ-cystathionase. Cysteine is further utilized for biosynthesis of GSH. The other two pathways that metabolize homocysteine re-synthesize methionine from homocysteine. One is catalyzed by methionine synthase (MS) and the other is catalyzed by betaine homocysteine methyltransferase (BHMT). Remethylation of homocysteine via MS requires 5-methyltetrahydrofolate (5-MTHF), which is derived from 5,10-methylenetetrahydrofolate (5,10-MTHF) in a reaction catalyzed by methylenetetrahydrofolate reductase (MTHFR). 5-MTHF is then converted to tetrahydrofolate (THF) as it donates its methyl group and THF is converted to 5,10-MTHF to complete the folate cycle. AdoMet is also utilized in the synthesis of polyamines, thereby generating methylthioadenosine (MTA) [9].

The liver is the main source of biosynthesis and consumption of the principle biological methyl donor, S-adenosylmethionine (AdoMet, also often abbreviated as SAMe and SAM). The synthesis of AdoMet from methionine and ATP is catalyzed by MAT isoenzymes. AdoMet is also a precursor for polyamine biosynthesis and in hepatocytes, a precursor for cysteine, the rate-limiting amino acid for the synthesis of the antioxidant glutathione (GSH) (Figure 1) [9]. Under normal physiological conditions, most of the AdoMet is utilized in transmethylation reactions and is converted to S-adenosylhomocysteine (AdoHcy, often abbreviated as SAH) (Figure 1) [10]. AdoHcy is a potent competitive inhibitor of transmethylation reactions. Both an increase in AdoHcy level and a decrease in the AdoMet:AdoHcy ratio are known to inhibit transmethylation reactions [9]. For this reason, the removal of AdoHcy is essential. The reaction that converts AdoHcy to homocysteine and adenosine is reversible and catalyzed by AdoHcy hydrolase [10]. The thermodynamics of this reaction favor the synthesis of AdoHcy. *In vivo*, the reaction proceeds in the direction of hydrolysis only if the products, adenosine and homocysteine, are rapidly removed [10].

In the liver, there are three pathways that metabolize homocysteine. One is the transsulfuration pathway, which converts homocysteine to cysteine via a two-step enzymatic process catalyzed by cystathionine β-synthetase (CBS) and γ-cystathionase, both requiring vitamin B_6_ [11]. The other two pathways in which homocysteine are metabolized lead to the re-synthesis of methionine. One reaction is catalyzed by methionine synthase (MS), which requires normal levels of folate and Vitamin B_12_. The other pathway is catalyzed by betaine homocysteine methyltransferase (BHMT), which requires betaine, a metabolite of choline [9,11]. Remethylation of homocysteine via MS requires 5-methyltetrahydrofolate (5-MTHF), which is derived from 5,10-methylenetetrahydrofolate (5,10-MTHF) in a reaction catalyzed by methylenetetrahydrofolate reductase (MTHFR). 5-MTHF is then converted to tetrahydrofolate (THF) as it donates its methyl group and THF is converted to 5,10-MTHF to complete the folate cycle. In the liver, AdoMet plays a regulatory role on methionine metabolism by inhibiting MTHFR and MS and activating CBS [9, 11]. Thus, when AdoMet is depleted, homocysteine is channeled to remethylation to regenerate AdoMet; whereas when AdoMet level is high, homocysteine is channeled to the transsulfuration pathway through AdoMet-mediated activation of CBS. Another contributing factor for this is that the K_m_ of CBS for AdoMet is 1-2.5 mM, whereas the K_m_ of MS for AdoMet is 60 μM [12]. Thus, when AdoMet level rises, it favors channeling of AdoMet to the transsulfuration pathway. AdoMet is also utilized in the biosynthesis of polyamines that are required for cell growth [9]. During the synthesis of polyamines, methylthioadenosine (MTA) is generated as a byproduct that is a known inhibitor of methylation [13].

## 3. Expression and Regulation of MAT Genes in Healthy and Diseased Liver

Mammalian systems express two MAT genes, namely *MAT1A* and *MAT2A* that encode the catalytic subunits of the enzyme (Table 1) [14]. The gene *MAT1A* encodes the α1 catalytic subunit, which organizes into dimers (MATIII) or tetramers (MATI) [14]. The gene *MAT2A* encodes for the α2 catalytic subunit found in the MATII isoform. A third gene, *MAT2B*, encodes for a β regulatory subunit that regulates the activity of MATII by lowering the inhibition constant (K_i_) for AdoMet and the Michaelis constant (K_m_) for methionine [15]. MAT1A is expressed mostly in adult liver [14,16] with low level of expression in extrahepatic tissues [17]. MAT1A is also expressed by pancreas with high level of expression seen in pancreatic acinar cells [17,18]. MAT2A is widely expressed in extrahepatic tissues and is also expressed in fetal liver but is replaced by MAT1A during development [16,19]. Different isoforms of MAT differ in kinetic and regulatory properties and sensitivities to inhibitors of MAT [9]. MAT II has the lowest K_m_ (∼4–10 μM), MAT I has intermediate K_m_ (23 μM–1 mM), and MAT III has the highest K_m_ (215 μM–7 mM) for methionine [20-22]. Although MAT isoenzymes catalyze the same reaction, they are differentially regulated by their product, AdoMet. AdoMet strongly inhibits MATII (50% inhibitory concentration (IC_50_) = 60 μM), which is close to the normal intracellular AdoMet concentration [10], whereas it minimally inhibits MATI (IC_50_ = 400 μM) and stimulates MATIII (up to eightfold at 500 μM AdoMet) [22]. Thus, the type of MAT isoform expressed in cells controls the steady state AdoMet level. Consistently we showed that hepatic cell lines over-expressing *MAT1A* have increased accumulation of AdoMet as compared to cells expressing *MAT2A* [23]. Increased expression of *MAT2B* can further lower steady state AdoMet level due to its influence on the K_i_ of MATII for AdoMet [24].

Adult differentiated liver expresses the *MAT1A*-encoded isoforms while increased hepatic expression of MAT2A is associated with increased growth, de-differentiation, and malignant degeneration [9,23]. Increased expression of MAT2B also provides a growth advantage to hepatoma cells, and although it is not expressed in normal liver, its expression is increased in liver cirrhosis and HCC [24].

*MAT1A* is silenced in HCC and during de-differentiation by both transcriptional and post-transcriptional mechanisms. *MAT1A* gene expression in the human hepatoblastoma cell line (HepG2) is regulated by histone acetylation and methylation. Treatment of these cells with demethylating agents or histone deacetylase inhibitors induces MAT1A mRNA expression and decreased MAT1A expression is associated with hypermethylation of a HpaII site at position -977 of the promoter [25]. Promoter hypermethylation also correlated with reduced *MAT1A* expression in cirrhotic patients and HCC [26]. Recently we reported that MAT1A 3′-UTR binds to the AU-rich RNA binding factor 1 (AUF1) [27]. AUF1 is one of the hnRNP proteins known to destabilize target mRNAs [28]. Interestingly we found that HCC specimens express higher AUF1 protein levels and knockdown of AUF1 increased MAT1A mRNA level [27]. AUF1 expression is also high in fetal liver and falls during liver development, which coincides with increased MAT1A expression [27].

MAT2A expression is also regulated at both transcriptional and post-transcriptional levels. We identified four *cis*-acting elements and *trans*-activating factors (Sp1, c-Myb, NFκB and AP-1) to participate in *MAT2A* transcriptional up-regulation in HCC [29,30]. We have also found promoter methylation and histone acetylation regulate the human *MAT2A* gene. The human MAT2A promoter is hypomethylated in HCC but hypermethylated in normal liver [31]. Histone acetylation status correlates with MAT2A expression in both human and rat so that a hyperacetylated status correlates with high MAT2A expression and *vice versa* [31,32]. More recently, we reported that MAT2A mRNA level is regulated by HuR and methylated HuR [27]. HuR is a ubiquitously expressed mRNA binding protein known to stabilize its target mRNAs, whereas methylated-HuR exerts the opposite effect [27]. Interestingly, during hepatocyte de-differentiation and in HCC, there is a switch from methylated-HuR to HuR binding to the 3′-UTR of MAT2A, resulting in increased MAT2A mRNA level [27]. AdoMet treatment results in higher methylated-HuR level, which contributes to its known inhibitory effect on MAT2A expression [33].

Relatively little is known about regulation of MAT2B expression. We recently reported that MAT2B has two dominant splicing variants, variant 1 (V1) and variant 2 (V2) [34]. Both variants are highly induced in HCC. Tumor necrosis factor α (TNFα) induces MAT2B V1 expression (but not V2) at the transcriptional level by mechanisms that involve AP-1 and NFκB [34]. Leptin increases while AdoMet inhibits MAT2B V1 promoter activity and expression by mechanisms that involve ERK and AKT signaling [35]. Whether MAT2B is regulated post-transcriptionally is unknown.

## 4. MAT Genes and HCC

Accumulating evidence support the notion that hepatic AdoMet deficiency is a risk factor in the development of HCC. Decrease in AdoMet content in preneoplastic and neoplastic liver may depend on changes in MAT isoenzyme pattern [36]. Fall in MAT1A expression and MATI/III activity with concomitant up-regulation of MAT2A occurs in hepatoma cell lines and rodent HCC as well as in human liver cirrhosis and HCC [37,38]. The MATII isoform is inhibited by its reaction product AdoMet so that its up-regulation does not lead to increase in AdoMet liver content [9]. Furthermore, MAT2B expression is induced in cirrhosis [24] so that most patients with chronic liver disease have hepatic AdoMet deficiency.

The *MAT1A* knockout (KO) mouse model was developed nearly 10 years ago to address how chronic AdoMet deficiency and deregulation of methionine metabolism may predispose to HCC. This model has provided invaluable insight into the pathogenesis of HCC in the setting of chronic AdoMet deficiency. This is highly relevant to human liver disease as the expression of MAT1A is markedly reduced [9]. *MAT1A* KO mice are viable since MAT1A is expressed shortly after birth and in the absence of MAT1A, MAT2A is induced [39]. Hepatic AdoMet level fell by nearly 75% and GSH level fell by 40%. At 3 months of age, *MAT1A* KO mice had body weights similar to wild-type littermates. However, their liver weights were significantly increased. At this age, the liver is histologically normal in the *MAT1A* KO mice fed a normal diet. Feeding a choline-deficient diet to the KO animals for six days induced severe macrovesicular steatosis compared to wild type controls. The livers of the 8-month-old wild-type littermates remained normal histologically, but the livers of 8-month old KO animals fed a normal diet exhibited macrovesicular steatosis involving 25–50% of hepatocytes and mononuclear cell infiltration, mainly in the periportal areas. By 18 months of age, many of the *MAT1A* KO mice developed liver cancer.

We have identified several mechanisms that can contribute to HCC development in the *MAT1A* KO mouse model. First is the existence of liver cancer stem cell population in the aging *MAT1A* KO mice. Methyl-deficient diets have been used to induce oval cell proliferation and HCC formation in susceptible models such as p53 knockout mice [40]. Oval cells are liver stem cells found in the non-parenchymal fraction of the liver and reside near the terminal bile ducts, at the hepatocyte-cholangiocyte interface. In normal adult liver, oval cells are quiescent and few in number and proliferate only during severe, prolonged liver injury and in various models of experimental carcinogenesis [41]. Our recent findings have shown that *MAT1A* KO mice have expansion of a population of oval cells that behave like liver cancer stem cells as they age [42]. These CD49f^+^ cells have markedly increased expression of several oncogenes such as K-ras and survivin. Moreover, a subpopulation of the CD49f^+^ cells that are also CD133^+^ possess tumorigenic potential when injected into immune deficient mice. This is the first demonstration of adult liver stem cells possessing tumorigenic potential without the use of a carcinogen or manipulation of tumor-suppressor or oncogene expression. Further work has shown that liver cancer stem cells from *MAT1A* KO mice possess highly enhanced mitogen-activated protein kinase (MAPK) signaling with increased level and activity of the extracellular signal regulated kinase (ERK) [43], known to be associated strongly with HCC development [44]. This is consistent with previous findings showing alterations in the MAPK pathway in *MAT1A* KO mice [36]. As compared to the CD133^-^ cell populations, CD133^+^ CD49f^+^ cells use their constitutive ERK activation to evade the apoptotic effect of transforming growth factor-β (TGF-β), a well-known growth inhibitor in hepatocytes [43,45].

Another mechanism is the role of genomic instability (GI). Differential expression of *MAT1A* and *MAT2A* genes can potentially influence DNA methylation and growth of human HCC [9,23]. DNA hypomethylation may generate GI during carcinogenesis [46]. The work of Calvisi *et al.* indicates that early changes in methionine/AdoMet metabolism and global DNA methylation may have a prognostic value for hepatocarcinogenesis in the majority of individuals, probably acting through a modulation of GI [47]. They have also shown that molecular alterations linked to AdoMet metabolism and DNA methylation are necessary for the development of the majority, but not all, human HCCs [47]. HCC demonstrates a high incidence of GI and the level of GI correlates with tumor stage [48]. The cellular defense pathway against GI includes several components, one of them being the Apurinic/Apyrimidinic Endonuclease 1 (APEX1), which is a multifunctional protein possessing both DNA repair and redox regulatory activities [49]. APEX1 is induced by oxidative stress and this is part of the defense mechanism against GI [50,51]. *MAT1A* KO mice exhibit increased oxidative stress and malignant transformation [36]. Based on this fact it would be expected that DNA repair pathways like APEX1 should be induced. On the contrary, it was observed that the expression of APEX1 was down-regulated in *MAT1A* KO mice and there was increased GI [52]. This decrease in APEX1 has been attributed to AdoMet deficiency in *MAT1A* KO mice. Primary hepatocytes placed in culture rapidly de-differentiate and show a decline in MAT1A expression and intracellular AdoMet level. Exogenous treatment of these cells with pharmacological doses of AdoMet prevents AdoMet depletion, blunts the fall in MAT1A expression, and importantly, stabilizes APEX1 protein [52]. Therefore AdoMet depletion can lead to decreased APEX1 protein stability and increased GI, contributing to malignant degeneration.

A third mechanism has to do with uncontrolled ERK activation. ERK activation is one of the several growth signals associated with highly malignant HCC phenotypes and it is tightly regulated in normal liver cells. One way through which ERK activity is kept under control is by the action of the dual-specificity MAPK phosphatase (DUSP1). DUSP1 is the first member of a family of dual-specificity MAPK phosphatases which can dephosphorylate both serine/threonine and tyrosine residues [53]. There is a reciprocal regulation between DUSP1 and ERK. Prolonged activation of ERK promotes phosphorylation at the Ser296 residue of its inhibitor, DUSP1 [54]. Phosphorylation of this specific residue renders the DUSP1 protein susceptible to proteasomal degradation by two substrate recognition protein belonging to a large S-phase kinase-associated protein/cullin/F box ubiquitin ligase: the S-phase kinase associated protein 2 (SKP2) and CDC28 protein kinase b1 complex. In contrast, transient activation of ERK leads to catalytic activation of DUSP1 followed by inactivation of ERK [54]. Thus DUSP1 feedback inhibits ERK and this activity of DUSP1 is crucial for the regulation of ERK activity in liver cells. In human HCC, DUSP1 expression is inversely correlated with proliferation index and microvessel density, and directly correlated with apoptotic index and survival [55]. Our group has shown that hepatic DUSP1 expression is low in *MAT1A* KO mice both at the mRNA and protein level, with protein level falling to lower level than mRNA [56]. Correcting AdoMet deficiency in *MAT1A* KO mice by exogenous AdoMet treatment normalized DUSP1 mRNA and protein levels [56]. AdoMet exerts its effect both transcriptionally and post-transcriptionally. AdoMet's ability to normalize DUSP1 mRNA level may be a p53-dependent effect because in *MAT1A* KO livers, binding of p53 to a p53 element in DUSP1 promoter was reduced compared to wild type livers and feeding AdoMet to these animals partially corrected p53 binding to the DUSP1 promoter. The increase in p53 binding in AdoMet fed animals is attributed to the fact that AdoMet stabilizes the APEX1 protein, which is a known transactivator of p53 [52]. The reason for the drastic drop in DUSP1 at the protein level in *MAT1A* KO mice is due to increased proteasomal activity, causing rapid degradation of the DUSP1 protein. Moreover *MAT1A* KO mice also have an increase in expression of SKP2 protein, an E3 ligase responsible for ubiquitination of DUSP1. This can further contribute to the decline in DUSP1 protein level. AdoMet appears to exert a direct effect on proteasomal activity as incubation of purified proteasomes with AdoMet led to increased degradation of some of its subunits and decreased proteasomal activity. Consistently, AdoMet treatment in *MAT1A* KO mice normalized proteasomal activity, increased DUSP1 protein level and reduced ERK activity back to baseline [56]. Thus, AdoMet deficiency predisposes to HCC by allowing for uncontrolled ERK activity due to decreased DUSP1 expression.

Another signaling pathway affected in the *MAT1A* KO mice is serine/threonine kinase 11 or LKB1, which lies upstream of AMP-activated protein kinase (AMPK). Although AMPK is primarily known for maintenance of energy homeostasis, we have shown that in hepatocytes, the most potent mitogen hepatocyte growth factor (HGF) exerts its mitogenic effect by activating LKB1 and AMPK and this can be blocked by AdoMet [57,58]. AMPK activation in hepatocytes led to nuclear to cytoplasmic HuR translocation, which stabilized several cyclin mRNAs to result in growth. In the presence of AdoMet, protein phosphatase 2A (PP2A) physically interacted with AMPK, leading to its dephosphorylation and inactivation [57]. Consistent with this, *MAT1A* KO mice have increased basal LKB1 and AMPK activity, cytoplasmic HuR level, increased cyclin D1 expression and basal proliferation [58]. AMPK can also activate eNOS, leading to increased nitric oxide (NO) formation. Interestingly, NO is known to inactivate MATI/III, resulting in lowering of AdoMet level which would release the inhibitory tone exerted on mitogens [58]. Consistently, HGF's mitogenic effect requires activation of LKB1, AMPK and eNOS and this cascade is activated during liver regeneration following 2/3 partial hepatectomy. Hepatic AdoMet level also falls early during liver regeneration and exogenous AdoMet treatment to prevent the fall in AdoMet level inhibited liver regeneration [58]. Most recently, we demonstrated that the increase in LKB1 activity in HCC cells derived from *MAT1A* KO livers is required for cell survival and that increased LKB1 activity is also found in human HCC samples [59]. Thus, contrary to the dogma that LKB1 serves as a tumor suppressor, it can facilitate growth and promote carcinogenesis in hepatocytes.

**Figure 2.**
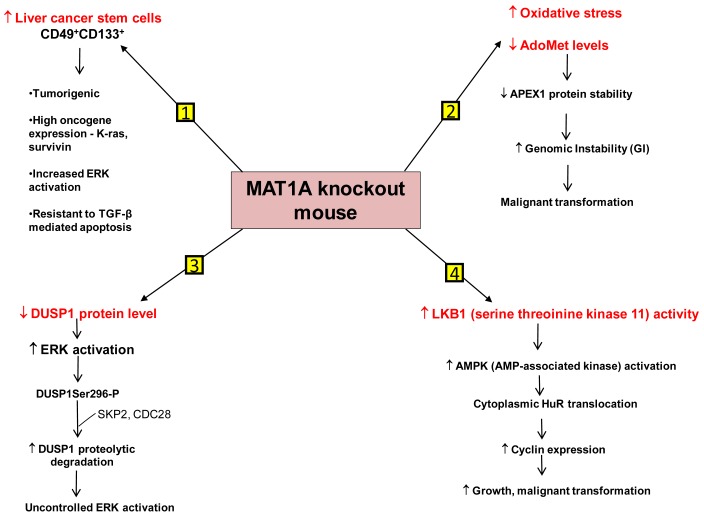
Mechanisms of HCC development in the *MAT1A* knockout mouse model. Several mechanisms have been recently elucidated that may promote the development of HCC in the *MAT1A* KO mice. These include: (**1**) Development of CD133^+^/CD49f^+^ liver cancer stem cells that have tumorigenic potential in nude mice, exhibit increased expression of certain oncogenes such as K-ras and survivin and are resistant to apoptosis mediated by TGF-β; (**2**) increased oxidative stress associated with a decrease in APEX1 protein stability and enhanced genomic instability (GI) leading to malignant transformation; (**3**) reduced DUSP1 expression, which allows ERK activity to go unchecked leading to phosphorylation of DUSP1 at Ser296 that further promote DUSP1 proteasomal degradation and uncontrolled ERK activation; (**4**) enhanced LKB1 activity leading to AMPK activation that further causes translocation of HuR protein from nucleus to cytoplasm. This leads to stabilization of cyclins thereby promoting growth and malignant transformation.

Figure 2 summarizes our current understanding of the mechanisms that predispose *MAT1A* KO mice to develop HCC.

So far, we have discussed the implications of reduced MAT1A expression, which is a differentiation marker for normal liver. Apart from a decline in MAT1A expression, HCC development is strongly associated with increased expression of the *MAT2A*-encoded isoform and its regulatory subunit encoded by the *MAT2B* gene. Studies on the effect of growth factors on MAT2A and MAT2B expression in the liver emphasize the important role played by these genes in proliferating liver. It is known that MAT2A expression is induced in the liver after partial hepatectomy. Latasa and colleagues showed that HGF strongly induces the expression of MAT2A in cultured rat hepatocytes by increasing the histone H4 acetylation associated with the MAT2A promoter [32]. Hepatic levels of HGF are markedly induced in partially hepatectomized livers and this results in activation of MAT2A [32], which in turn is required for HGF's mitogenic response [60]. Another growth factor known to promote growth and invasive potential of liver cancer cells is the product of the obese (Ob) gene, leptin [61,35]. Recent work in our laboratory has shown that leptin induced *MAT2A* and *MAT2B* genes in HepG2 cells and silencing of either *MAT2A* or *MAT2B* genes blocked the mitogenic potential of leptin [35]. MAT2A facilitates leptin-mediated growth by raising intracellular AdoMet level to promote polyamine biosynthesis. MAT2B on the other hand plays a novel role in promoting leptin signaling. Gene silencing studies have shown that MAT2B is required for ERK and PI3-K activation that are important in the leptin-mediated growth signaling pathway. Also, MAT2B appears to be required for STAT3 activation, an upstream event in leptin signaling [35]. These findings suggest that apart from its role as a regulatory subunit of MATII, MAT2B is involved in multiple growth-associated signaling functions in the cell. The two major variants of MAT2B, V1 and V2 that are highly induced in HCC are differentially regulated by TNF-α [34]. TNF-α is a pleiotropic cytokine that induces cellular response such as proliferation and cell death. It is known to induce apoptosis by a JNK-mediated mechanism [62]. In normal hepatocytes, TNF-α has no cytotoxic effect because NF-κB is also induced to protect from TNF-α-mediated apoptosis by inducing the expression of protective genes against apoptotic cell death [62]. One of these protective genes in liver cancer cells is *MAT2B V1*. Indeed, silencing *V1* but not *V2* sensitizes HepG2 cells to TNF-α induced apoptosis. Hence, MAT2B V1 acts as a NF-κB-dependent survival factor in liver cancer cells. In order to delineate targets of MAT2B in liver cancer cells, our laboratory employed a proteomics approach to identify novel binding partners for MAT2B variants [63]. The findings from this work showed that both MAT2B variants are localized in the nucleus as well as the cytoplasm of HepG2 cells. In these cells MAT2B V1 and V2 can interact with HuR, a mRNA binding protein that is known to stabilize mRNA for cyclins [63]. Moreover, both variants can modulate the subcellular localization of HuR. In unstimulated cells, HuR is predominantly nuclear but becomes cytoplasmic in response to proliferative and stress stimuli [64]. Over-expression of MAT2B V1 or V2 increases the cytoplasmic content of HuR and induces the expression of downstream targets of HuR, namely cyclin D1 and cyclin A. Hence one of the mechanisms by which MAT2B variants promote survival of liver cancer cells is by modulating HuR-mediated interactions with factors implicated in cell growth.

## 5. MAT Genes as Therapeutic Targets for HCC

AdoMet was examined in the chemoprevention of HCC over 20 years ago. This was based on the rationale that models of hepatocarcinogenesis exhibit decreased hepatic AdoMet levels, global DNA hypomethylation, increased susceptibility to GI and enhanced proliferation rate. Garcea *et al.* have shown that livers of rats treated with diethylnitrosamine develop actively remodeling, preneoplastic nodules after one week [65]. These nodules grow actively after 11 weeks and are resistant to remodeling but do not lead to cancer. These were named persistent nodules. The nodules exhibited low AdoMet/AdoHcy ratios, induction of c-myc, c-Ha-ras and c-Ki-ras and hypomethylation of DNA. AdoMet administration from 3-11 weeks in these rats decreased the size and number of these nodules. AdoMet treatment also reduced the expression of c-myc, c-Ha-ras and c-Ki-ras and prevented the decrease in AdoMet/AdoHcy ratio and hypomethylation [65]. Prolonged AdoMet administration in rats also prevents initiated preneoplastic cells from progressing to cancer [66]. To understand whether AdoMet can be used in a HCC model where liver AdoMet level is unchanged and whether AdoMet can be effective as a chemotherapeutic agent in established HCC, we used a rat model of liver cancer in which the aggressively growing human hepatoma cell line, H4IIE is injected directly into the liver parenchyma of normal rats [67]. After two weeks, the animals develop tumors in the liver. Continuous intravenous AdoMet infusion into these rats immediately after tumor cell injection inhibited HCC formation. However AdoMet infusion for 24 days did not affect the size of already established tumors. In the established tumors, even though plasma AdoMet levels were increased, hepatic AdoMet levels were minimally affected (30% higher). This is in contrast to 10-fold higher hepatic AdoMet levels after a 24 hour infusion. This is because chronic AdoMet administration led to a compensatory induction in hepatic glycine N-methyltransferase (GNMT) expression, which prevented AdoMet accumulation. Therefore, in established tumors, the level of AdoMet required to trigger tumor cell apoptosis and inhibit growth was not reached. However, whether AdoMet treatment in patients with HCC would be effective remains to be examined because GNMT is silenced in most HCC [67].

One plausible explanation for AdoMet to inhibit the establishment of HCC is its selective pro-apoptotic effect against liver cancer cells [9] during the acute setting, prior to the compensatory response of the methyltransferases. Another potential way in which AdoMet prevents HCC development is by influencing angiogenesis. Microarray analysis showed that AdoMet can inhibit the expression of pro-angiogenic factors such as platelet-derived growth factor-alpha (PDGF-α) and midkine. It can also induce the expression of proteins like Type XVIII collagen, which is a precursor for the anti-angiogenic peptide, endostatin [67]. Thus, it remains potentially an ideal agent for HCC chemoprevention in man.

High tissue AdoMet level may be needed to exert a pro-apoptotic and anti-angiogenic effect on cancer cells. Due to the compensatory response of methyltransferases described above, AdoMet is not a very effective chemotherapeutic agent when GNMT expression is normal. Recently, we have tried to overcome this problem by forcefully expressing the *MAT1A* gene in tumors generated in nude mice with the hope that this would raise intracellular AdoMet levels and hence inhibit tumorigenesis [68]. Human hepatoma cell line Huh7 was stably transfected with *MAT1A* expression vector. The Huh7 cells stably over-expressing *MAT1A* were injected into nude mice to generate tumors. Compared to control cells, *MAT1A* transfectant generated tumors were smaller in size and weight and had higher intracellular AdoMet levels. Consequently, cell growth was inhibited in *MAT1A* transfectants compared to control tumors. Microarray analysis of *MAT1A* over-expressing tumors provided further insight into how this gene affects cell growth, apoptosis and angiogenic pathways. The findings clearly indicated that *MAT1A* over-expression inhibited ERK signaling pathway with a concomitant induction in the apoptotic protein, caspase 7. This is one plausible mechanism for the decreased growth and increased apoptosis in *MAT1A* transfectants. Another important target of *MAT1A* identified from microarray analysis is spp1 or osteopontin, an important cell growth and angiogenesis factor in HCC [69]. Binding of spp1 to integrin receptors in cancer cells leads to activation of the ERK and PI3-K survival signaling pathways causing enhanced cell growth [69]. *MAT1A* transfected tumors exhibited reduced expression of spp1. This is consistent with our finding that forced expression of MAT1A affects downstream pathways of spp1 signaling, namely ERK and PI3-K activation. *MAT1A* over-expression also induced PP2A, a phosphatase that keeps ERK and the effector of PI3-K signaling, AKT, in dephosphorylated state [70]. This is another putative mechanism by which *MAT1A* overe-xpression lowers ERK and AKT activity leading to reduced growth.

While chronic hepatic AdoMet depletion clearly predisposes the organ to malignant degeneration, it should be noted that mice lacking GNMT have markedly elevated hepatic AdoMet levels and they also develop HCC [71]. However, the mechanism for HCC in this KO model is different from the *MAT1A* KO mouse model. Increased AdoMet levels resulted in aberrant DNA hypermethylation of inhibitors of the Ras and JAK/STAT pathway [71]. Activation of Ras/MEK/ ERK and JAK/STAT signaling pathways are thought to be essential for human HCC development, and that suppression of Ras and JAK/STAT inhibitors (such as RASSF1, CIS and SOCS 1-3) may be responsible for the persistent activation of these pathways [72]. Consistent with this, Ras and downstream effectors of Ras involved in proliferation and survival, including pRaf, pMEK1/2, and pERK1/2, and activation of JAK/STAT and their downstream targets such as pJAK1/2, pSTAT1/STAT3, cyclin D1, cyclin D2 and Bcl-xL were all increased in HCCs of GNMT KO mice. Importantly, the expression of two Ras inhibitors (RASSF1 and 4) and four JAK/STAT inhibitors (SOCS1, 2 and 3, and CIS) was reduced in the HCCs from GNMT KO mice [71].

Recently, Reytor *et al.* [17] have shown that the MAT1A protein exhibits two partially overlapping areas at its C-terminal end that are involved in cytoplasmic retention and nuclear localization. By the use of mutants of this region of the protein, the authors have shown that both tetrameric and monomeric forms of the MATI/III catalytic subunits are localized to the nucleus. The authors speculate that in this subcellular compartment the active, oligomeric forms might be responsible for AdoMet synthesis. This is supported by the fact that nuclear accumulation of the active MAT1A protein correlated with higher levels of histone H3K27 trimethylation, an epigenetic modification associated with gene repression, and DNA methylation. Cells transfected with specific mutants K368A and K369A localized the MAT1A subunits primarily in the nucleus and these nuclear mutants exhibited higher levels of H3K27 methylation as compared to wild type MAT1A. Since AdoMet is highly unstable, its *in situ* synthesis for gene repression could be guaranteed by the localization of active MAT1A forms in the nucleus of cells. Based on these findings, it would be worthwhile to examine the endogenous nuclear localization of MAT1A and MAT2A-encoded proteins in HCC cells and understand whether intra-nuclear levels of these proteins in HCC affects the nuclear AdoMet levels and whether this is associated with changes in histone or DNA methylation that could influence HCC development.

Reduction in MAT2A or MAT2B variants can also be used as a strategy for HCC. Exogenous AdoMet blocks MAT2A and MAT2B expression and this is one of the ways in which AdoMet can be chemopreventive in the H4IIE tumor model described above [67] but the compensatory response against excess AdoMet limits its use as a chemotherapeutic agent. Studies on the use of specific RNAi against MAT2A or MAT2B genes are limited to *in vitro* analysis in human liver cancer cell lines. Our group has investigated the effect of silencing either MAT2A or MAT2B in HepG2 cells. Results show that knockdown of either gene inhibits proliferation of liver cancer cells in response to mitogens like leptin [35]. Whether *in vivo* knockdown of MAT2A or MAT2B can be effective in treating HCC cases that exhibit over-expression of these genes is an area that warrants future investigation.

## 6. Conclusions

MAT genes are required for the biosynthesis of AdoMet. AdoMet is the principal biological methyl donor that is essential for cell survival. Of the two MAT genes synthesizing AdoMet, *MAT1A* is highly expressed in adult liver and pancreas with low level of expression in other tissues but *MAT2A* is widely expressed in extrahepatic tissue. Another gene *MAT2B* encodes a regulatory subunit of the MAT2A-encoded isoenzyme. In HCC *MAT1A* is silenced whereas *MAT2A* and *MAT2B* are induced. Studies in the *MAT1A* KO mouse model have shed light on the mechanisms of how *MAT1A* silencing and consequent AdoMet depletion can lead to HCC. These include the existence of liver cancer stem cells, the de-regulation of cell growth pathways like ERK and LKB1/AMPK cascades and the alteration of DNA repair genes. MAT2A and MAT2B induction in HCC are also linked to essential growth signals in the cell. Growth factors like HGF and leptin induce MAT2A and silencing of *MAT2A* blocks the proliferative effect of these mitogens. *MAT2B* silencing also blocks the proliferative effect of mitogens. MAT2B promotes growth signaling by influencing cell survival pathways, ERK and PI3-K. In essence, alterations in MAT genes and AdoMet biosynthesis in the injured liver can severely interfere with the regulation exerted by these genes on cell growth. This lack of control is an important factor in the development of HCC. Replenishing endogenous AdoMet pool may be effective in chemopreventing HCC, whereas forced expression of *MAT1A* may be effective in treating already existing HCC. Another strategy is to silence *MAT2A* or *MAT2B* in cases of HCC that exhibit enhanced expression of these genes. Finally, an intriguing area that warrants further study is whether there is abnormal nuclear expression of MAT1A and MAT2A-encoded proteins in HCC and whether this favors growth by epigenetic mechanisms.

## Figures and Tables

**Table 1.** MAT genes, isoenzymes, kinetics and expression patterns.

**Gene**	**MAT Isoform**	**Catalytic Subunit**	**Regulatory Subunit**	**Km for Methionine**	**Expression**		**Ref.**

					**Healthy Liver**	**HCC**	
MAT1A	MATI	α1 (tetramer)		23 μM–1 mM	Abundant	Decreased	[14-16,37,38]
MAT1A	MATIII	α1 (dimer)		215 μM–7 mM	Abundant	Decreased	[14-16,37,38]
MAT2A	MATII	α2		4-10 μM	Low	Increased	[14-16,37,38]
MAT2B			B		Low	Increased	[14-16,24,34]
